# A founder *CEP120* mutation in Jeune asphyxiating thoracic dystrophy expands the role of centriolar proteins in skeletal ciliopathies

**DOI:** 10.1093/hmg/ddu555

**Published:** 2014-10-30

**Authors:** Ranad Shaheen, Miriam Schmidts, Eissa Faqeih, Amal Hashem, Ekkehart Lausch, Isabel Holder, Andrea Superti-Furga, UK10K Consortium, Hannah M. Mitchison, Agaadir Almoisheer, Rana Alamro, Tarfa Alshiddi, Fatma Alzahrani, Philip L. Beales, Fowzan S. Alkuraya

**Affiliations:** 1 Department ofGenetics, King Faisal Specialist Hospital and Research Center, Riyadh 11211, Saudi Arabia,; 2 Genetics and Genomic Medicine, UCL Institute of Child Health, 30 Guilford Street, London WC1N 1EH, UK,; 3 Department of Pediatrics, King Fahad Medical City, Riyadh 59046, Saudi Arabia,; 4 Department of Pediatrics, Prince Sultan Military Medical City, Riyadh 11159, Saudi Arabia,; 5 Pediatric Genetics Division, Center for Adolescent and Pediatric Medicine, University Hospital Freiburg, Freiburg 79108, Germany,; 6 Department of Pediatrics, Lausanne University Hospital, University of Lausanne, Lausanne 1011, Switzerland,; 7 Department of Anatomy and Cell Biology, College of Medicine, Alfaisal University, Riyadh 11533, Saudi Arabia; 8 Department of Human Genetics, Radboud University Medical Center and Radboud Institute for Molecular Life Sciences, Radboud University Nijmegen, 6500 HB Nijmegen, the Netherlands

## Abstract

Jeune asphyxiating thoracic dystrophy (JATD) is a skeletal dysplasia characterized by a small thoracic cage and a range of skeletal and extra-skeletal anomalies. JATD is genetically heterogeneous with at least nine genes identified, all encoding ciliary proteins, hence the classification of JATD as a skeletal ciliopathy. Consistent with the observation that the heterogeneous molecular basis of JATD has not been fully determined yet, we have identified two consanguineous Saudi families segregating JATD who share a single identical ancestral homozygous haplotype among the affected members. Whole-exome sequencing revealed a single novel variant within the disease haplotype in *CEP120*, which encodes a core centriolar protein. Subsequent targeted sequencing of *CEP120* in Saudi and European JATD cohorts identified two additional families with the same missense mutation. Combining the four families in linkage analysis confirmed a significant genome-wide linkage signal at the *CEP120* locus. This missense change alters a highly conserved amino acid within CEP120 (p.Ala199Pro). In addition, we show marked reduction of cilia and abnormal number of centrioles in fibroblasts from one affected individual. Inhibition of the *CEP120* ortholog in zebrafish produced pleiotropic phenotypes characteristic of cilia defects including abnormal body curvature, hydrocephalus, otolith defects and abnormal renal, head and craniofacial development. We also demonstrate that in *CEP120* morphants, cilia are shortened in the neural tube and disorganized in the pronephros. These results are consistent with aberrant CEP120 being implicated in the pathogenesis of JATD and expand the role of centriolar proteins in skeletal ciliopathies.

## Introduction

Jeune asphyxiating thoracic dystrophy (JATD [MIM208500]) is a very rare skeletal dysplasia affecting 1:100 000 live births (1). The core phenotype comprises a small or narrow thoracic cage, often leading to early lethality by virtue of cardiorespiratory insufficiency, and characteristic spurs (trident) of the acetabular roof. Other skeletal findings include short long bones as well as polydactyly (2). This skeletal profile is shared by other skeletal dysplasias that are collectively known as short rib-polydactyly (SRP [MIM 208500, 611263, 613819, 615630, 266920, 615633, 613091, 269860, 614091, 615503]) syndromes and are differentiated clinically by the degree of severity of skeletal changes as well as the presence of extra-skeletal anomalies (2). However, the value of this distinction is questionable in view of the significant overlap in their associated clinical spectrums and, more importantly, in their molecular pathogenesis (see below) (3).

Both the skeletal as well as the extra-skeletal features of JATD are known to exist in a number of malformation syndromes referred to as ciliopathies because the underlying pathogenesis revolves around impaired physiology of a microtubule-based cellular antenna known as the primary cilium (4). For example, JATD individuals can have a cerebellar hypoplasia pattern typical of Joubert syndrome (5), abnormal retina typical of cilia-related retinal dystrophy (6) and cystic renal changes typical of nephronophthisis (7,8). These clinical observations have been corroborated over the past few years by the demonstration that JATD can be caused by mutations in genes that encode various ciliary proteins, which justifies the classification of JATD as a skeletal ciliopathy (2).

To date, nine genes have been found to be mutated in individuals with JATD (Supplementary Material, Fig. S1). The most frequently mutated gene appears to be *DYNC2H1* (MIM 603297), which encodes a component of the dynein motor involved in the retrograde transport of cargo from the tip of the cilium to its base. Two studies have shown that *DYNC2H1* is mutated in 41–59% of JATD individuals (3,9). Several components of the ciliary cargo transport system known as intraflagellar transport (IFT) were also implicated in the pathogenesis of JATD including *IFT80* (MIM 611177) (10), *TTC21B*/*IFT139* (MIM 612014) (11), *IFT140* (MIM 614620) (12), *WDR19*/*IFT144* (MIM 608151) (13), *WDR34* (MIM 613363) (14), *WDR60* (MIM 615462) (15), and *IFT172* (MIM 607386) (8). Further, mutations in *CSPP1* (MIM 611654) have been recently identified in individuals with Jeune Syndrome and Joubert phenotype (16,17). Importantly, several of these genes have been found to be mutated across the phenotypic spectrum of SRP syndromes, lending further support to the notion that the clinical distinction is arbitrary (18,19). Despite the recent expansion of locus heterogeneity of JATD, it is clear that not all affected individuals can be explained by mutations in these genes and that additional loci likely exist. In this report, we establish a novel locus for JATD on 5q23.2 by linkage analysis and demonstrate that a mutation in *CEP120* (MIM 613446) within this locus is the most likely cause of the disease.

## Results

### JATD is mapped to *CEP120*

**Figure 1.**
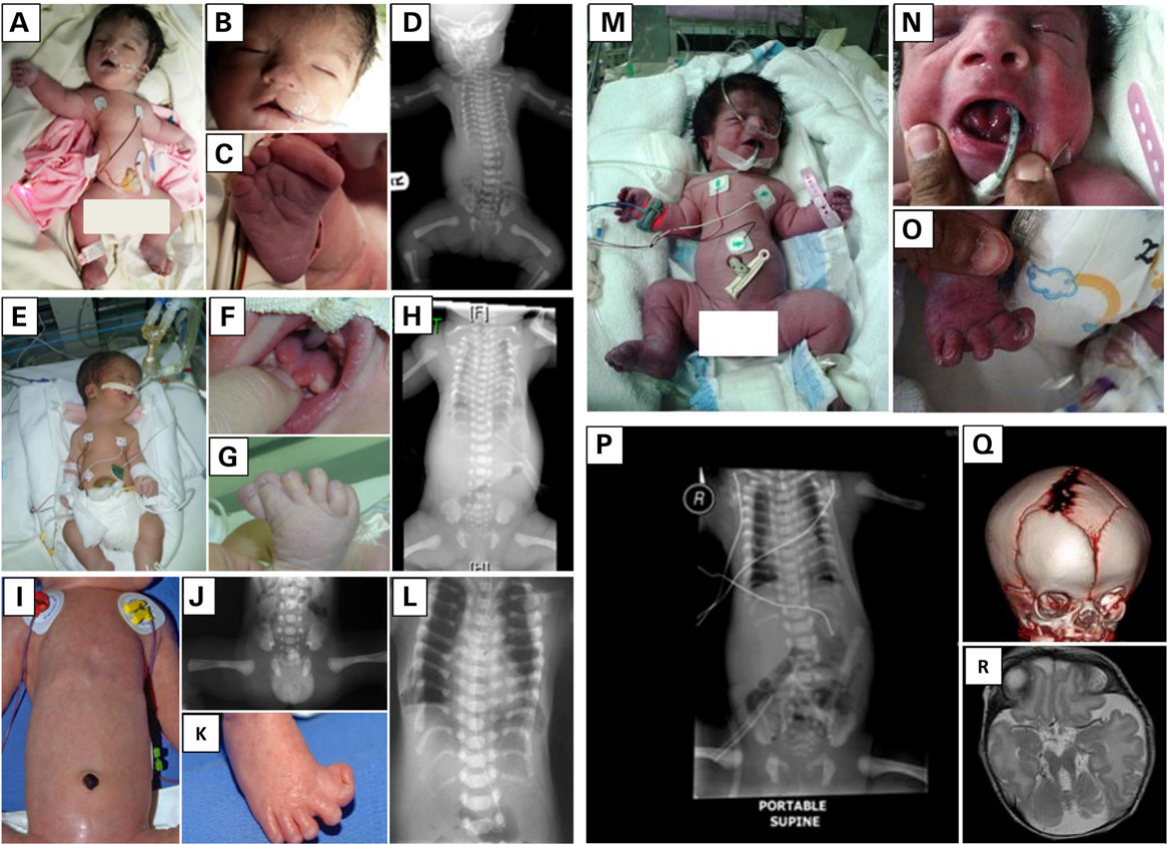
Clinical images of the study families. (**A**–**D**) Postnatal clinical photograph and radiological imaging of the index case from Family 1 showing severely narrow chest and thorax, mild cleft lip (notch), short limbs and synpolydactyly. (**E**–**H**) Postnatal clinical photograph and radiological imaging of the index case from Family 2 showing very narrow chest, short extremities, synpolydactyly, tongue hamartoma (lobulated tongue) and omphalocele. (**I**–**L**) Postnatal clinical photograph and radiological imaging of the individual Family 3_II:1 showing long and narrow thorax with short horizontal ribs, dysplastic pelvis with acetabular spurs and hexadactyly of the feet. (**M–R**) Postnatal clinical photograph, radiological imaging, 3D skull computed tomography and MRI of the index case from Family 4 showing coarse facies, midface hypoplasia, partially bifid tongue, polydactyly, short tubular bones, bell-shaped thorax with short ribs, unilateral coronal craniosynostosis with prominent and widened anterior and posterior fontanels, and brain vermian hypoplasia with molar-tooth appearance.

**Figure 2.**
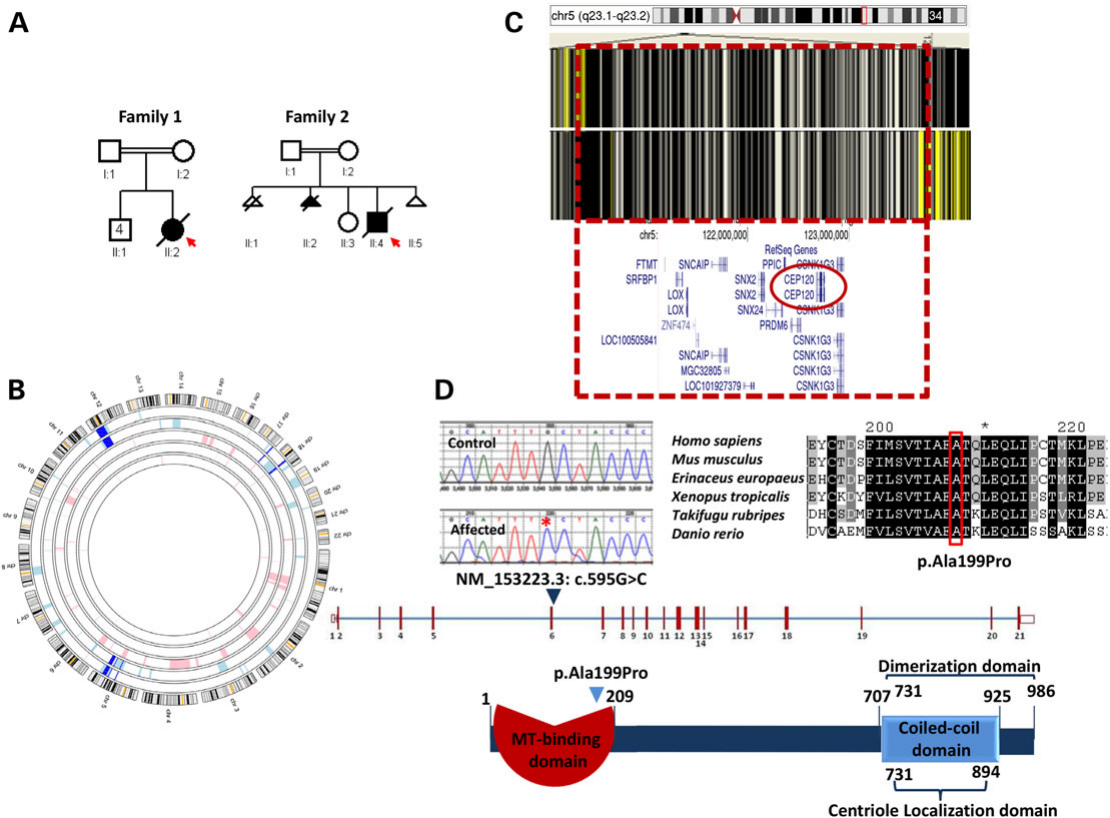
Identification of homozygous missense variant in *CEP120* in two consanguineous Saudi families with JATD. (**A**) Pedigrees of the two consanguineous Saudi families. The index is indicated in each pedigree by an arrow. (**B**) AgileMultiIdeogram showing the shared ROH regions of homozygosity shared between the index cases from each of the two families (dark blue). (**C**) AutoSNPa showing the identical haplotype between individual II:2 in Family 1 and individual II:4 in Family 2 denoted by black lines (boxed in red lines). (**D**) Upper panel: sequence chromatogram of the missense mutation (control tracing is shown for comparison, and the location of the mutation is denoted by an asterisk) and multisequence alignment orthologs of the mutation (p.Ala199) residue showing that the mutation is conserved across species down to *Danio rerio* (boxed in red). Lower panel: schematic representation of CEP120 and the location of the homozygous missense substitution identified in this study.

The first individual (Family 1_II:2) is a female stillbirth who was born vaginally at 35 weeks of gestation to healthy first-cousin Saudi parents. In addition to a severely narrow chest, apparent physical anomalies included relative macrocephaly, hypertelorism, scant eyebrows, a mild cleft lip (notch), short limbs and synpolydactyly (Figs 1A–D and 2A). Radiological examination revealed a narrow thorax with very short and horizontal ribs, a relatively normal spine, dysplastic and small pelvic bones with a narrow sciatic notch bilaterally, round ended femur bones, small tibia and fibula, pre-axial polydactyly and extremely hypoplastic middle and distal phalanges. She died at 5 h of age due to severe cardiopulmonary insufficiency and autopsy was declined. The second individual (Family 2_II:4) is a male baby born to healthy first-cousin Saudi parents who have a healthy child but had an intrauterine fetal death with skeletal dysplasia and pre-axial polydactyly. The similarity with the index case in Family 1 was striking in that there was a very narrow chest, short extremities and synpolydactyly. However, he lacked cleft lip but instead had tongue hamartoma (lobulated tongue) and omphalocele. His skeletal radiological findings included small facial bones, narrow thorax with small and horizontal ribs, flaring of the iliac bones with trident sciatic notch, short long bones of the upper and lower limbs and pre-axial polydactyly. Brain magnetic resonance imaging (MRI) showed cerebellar hypoplasia with Dandy-Walker malformation and extra-axial fluid collection. Abdominal ultrasound was normal as was electroretinography (ERG; Figs 1E–H and 2A). He later succumbed to cardiopulmonary insufficiency. Again, the family declined autopsy.

In order to identify the underlying genetic cause, DNA from lymphocytes was extracted from the two affected individuals as well as their parents and available healthy siblings after enrolling them in an institutional review board (IRB)-approved protocol with informed consent. The two families were apparently unrelated so we set out to perform single-nucleotide polymorphism (SNP)-based autozygome analysis in each index as described before (20). However, it was later apparent that the autozygome of the two individuals overlapped on six chromosomal locations (Fig. 2B, Supplementary Material, Table S1), none of which is known to harbor genes previously reported to be mutated in JATD. More importantly, only one of these overlaps represented an identical shared haplotype (Fig. 2C and Supplementary Material, Table S1) so we hypothesized that JATD in these two families is caused by a recessive mutation contained within this shared founder haplotype. The critical locus (chr5:121,129,268–123,737,922) harbors 17 RefSeq genes (Fig. 2B) so we proceeded with whole-exome sequencing (WES) followed by filtering the variants as described before (21). Briefly, we only considered homozygous coding/splicing variants within the 2.6 Mb critical locus. Only one novel variant fitting these criteria was identified (NM_153223.3: c.595G>C). This variant which was confirmed by Sanger sequencing and is predicted to replace a highly conserved amino acid residue in CEP120, p.Ala199Pro (Fig. 2D), is predicted to be pathogenic in silico [Polyphen = probably damaging (1), SIFT = deleterious (0.01) and GERP = 5.75] and is absent in the 1000 Genomes database. This allele was observed once in the heterozygous state among 1294 Saudi alleles tested and twice among the 12 000 alleles tested in the exome variant server. The very low frequency (MAF 0.00023) is compatible with this mutation being causal of a rare phenotype like JATD with a disease prevalence of less than 1:100 000. Reassuringly, no pathogenic variants were identified in any of the genes previously reported to be mutated in JATD and we achieved 100% coverage of the coding exons of all the genes within the critical locus by WES with an average depth of 45× and a range of 3–131.

In order to test whether the variant we identified in *CEP120* is disease-causing, we sought to search for likely pathogenic variants in this gene in independent individuals. A total of 109 affected individuals of JATD/SRPS (representing Saudi and European cohorts) were analyzed by exome sequencing, autozygosity mapping and/or Sanger sequencing of *CEP120.* This ‘replication’ cohort proved complementary in that two additional families (Family 3 and Family 4) were found to carry the same CEP120 variant described above. Reassuringly, genome-wide linkage analysis combining the four families revealed a single linkage peak on 5q23.2 with logarithm of odds score of 3.6 (Supplementary Material, Fig. S2).

The index individual in Family 3 (Family 3_II:1; Supplementary Material, Fig. S3) is the first child of healthy unrelated Swiss parents; however, both parents come from the same village. He was born at term after an unremarkable pregnancy with a birth weight of 2.8 kg (10th centile), body length of 45 cm (<3rd centile) and occipitofrontal head circumference of 32 cm (25th centile). At birth, thoracic dystrophy and symmetric pre-axial hexadactyly of both feet was evident; the hands were normal. Except for microretrognathia, no facial dysmorphism or cleft lip was noted. Respiratory insufficiency necessitated intubation and high frequency mechanical ventilation from birth. Because of hypoplastic lungs, oxygenation was insufficient and the clinical course was unfavorable with fatal pulmonary failure in the first week of life. Ultrasound scans showed decreased size of both kidneys with increased echogenicity; the liver was unremarkable. No postmortem examination was performed. Haplotype analysis using intragenic SNPs between Family 1_II:2, Family 2_II:4 and Family 3_II:1 indicated that the mutation resulted from a common founder individual with shared runs of homozygosity (ROH) of 714 139 bps, most likely very ancient consistent with the different ethnic and geographic origin of these families (Supplementary Material, Fig. S2).

The index in Family 4 (Family 4_II:1; Supplementary Material, Fig. S3) was born via C/S section at 37 weeks of gestation due to complications of uncontrolled gestational diabetes mellitus. Family history is notable for first cousin parents who had four consecutive miscarriages and a boy with history of renal tumor. Short stature was evident with length of only 35 cm, as was the very narrow chest and associated respiratory distress requiring neonatal intensive care unit admission and ventilator support. Echocardiography revealed large patent ductus arteriosus. He had coarse facies, midface hypoplasia, large nose with prominent nostrils, partially bifid tongue, natal teeth and large ears with prominent lobules. The limbs were micromelic with pre-axial polydactyly in all extremities and hallux deformity bilaterally. The genitalia was ambiguous with phallus-like structure and prominent prepuce. Routine karyotype demonstrated normal 46;XY and abdominal and pelvic sonography showed undescended testicles. Endocrine workup was normal. The skeletal survey showed short tubular bones, bell-shaped thorax with short ribs and acetabular bony spurs with resulting narrow sacrosciatic notch. The iliac wings are small and squared and the acetabular roof is horizontal. A three-dimensional (3D) skull computed tomography revealed unilateral coronal craniosynostosis with prominent and widened anterior and posterior fontanels. Brain MRI revealed vermian hypoplasia with molar-tooth appearance. The patient died on the seventh day because of poor lung function despite high ventilator support (Fig. 1). The DNA of the index was not available for testing, but his parents were found to harbor the same CEP120 mutation on the same haplotype background as families 1–3 in a heterozygous state (Supplementary Material, Fig. S2).

### *CEP120* mutation causes abnormal cilium number and aberrant centrosome number

*CEP120* encodes a protein that was previously identified by two independent functional screens. The first involved global proteomics analysis of purified centrosomes in which *CEP120* was found to encode a generic coiled-coil domain containing protein dubbed CCDC100 (22). The second was a yeast-two-hybrid screen for interactors with focal adhesion kinase (FAK), which is known to play a role in organizing centrosome-associated microtubules. This screen uncovered a 120 kD protein shown to localize to the centrosome in the same study, so it was named centrosomal protein of 120 kD (CEP120) (23). The centrosomal localization of CEP120 was found to be resistant to microtubule destabilization, which is consistent with it representing a ‘core’ centriolar protein as later shown by demonstrating that it localizes to the entire ‘barrel’ of the centriole but not its lumen (24). Centrosomes are formed of a pair of microtubule-based mother and daughter centrioles and a surrounding matrix (pericentriolar matrix or PCM). These cellular organelles serve numerous roles that vary depending on the cell cycle. In dividing cells, centrosomes assume the role of the microtubule-organizing center (MOC) in animal cells and serve the critical function of organizing the mitotic spindle to ensure proper alignment and disjunction of chromosomes (in mitosis) and chromatids (in meiosis). In non-dividing cells, however, the mother centriole assumes the role of basal bodies that nucleate the axoneme of the budding cilium (25).

**Figure 3.**
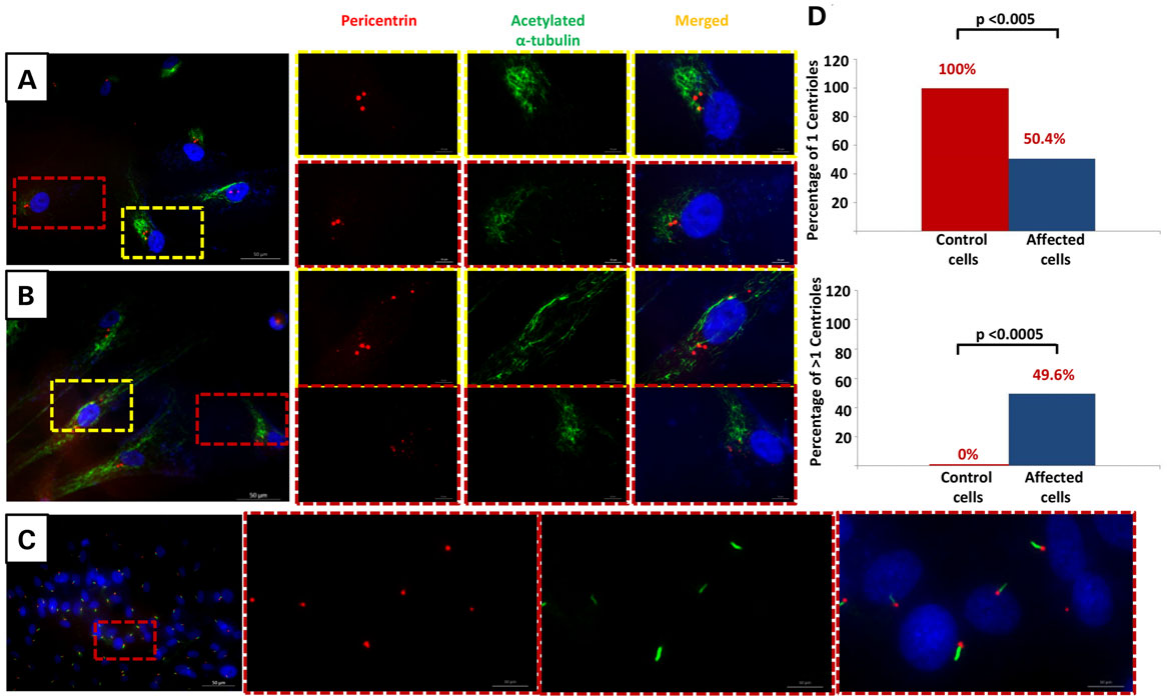
CEP120-related ciliopathy is associated with decreased cilium number and abnormal number of centrosomes. Immunofluorescence images of serum-starved fibroblasts from individual II:2 in Family 1 (**A** and **B**) and control fibroblasts (**C**) stained for pericentrin (Abcam) (red), the ciliary marker acetylated a-tubulin (Sigma-Aldrich) (green) and DNA (blue). Compared with control, fibroblasts from II:2 in Family 1 showed a marked decrease in cilium number and variable number of centrosomes (some cells have 3, 4 and fragmentation of more than 5). Scale bars in the main figure: 50 µm. (**D**) Graphs show significant reduction in the number of fibroblast cells derived from individual II.2 from Family 1 with one centrosome (*P*< 0.005) and an increase in number of cells with more than one centrosome (*P* < 0.0005).

Although deficiency of CEP120 has been found to be compatible with centrosome formation, it has been suggested that normal levels of CEP120 are required for the proper elongation and division of the centrioles. Depletion of CEP120 resulted in shorter centrioles whereas overexpression was associated with centrioles that are longer than average (26). Similarly, depletion of CEP120 has been associated with failure of centrioles to duplicate resulting in loss of centrioles with progressive cell divisions, and although overexpression was shown by some to result in an amplification phenotype, others failed to replicate this (24). Because all JATD-related genes to date have been linked to ciliary defects, we sought to investigate the potential effect of the JATD-associated *CEP120* mutation on centrosomes and decreased cilium number. We carried out a standard ciliogenesis assay on skin-derived fibroblasts from individual Family 1-II:2 and observed a dramatic reduction in the frequency of ciliated cells compared with control (three biological replicates, 87% of the cells without cilia in patient cells versus 7% in controls; *P* < 1.977 × 10^−8^). The assay was based on using two axonemal markers (acetylated α-tubulin and IFT88) and cilia formation was counted based on observing protrusion of the cilia axoneme. No multiciliated cells were observed. Furthermore, in contrast to control fibroblast cells after 24 h of serum starvation to induce ciliogenesis serum starvation that consistently showed a single centrosome, about half the cells derived from individual Family 1_II:2 showed a variable number of centrosomes ranging from 1 to >4 (Fig. 3A, B, D and Supplementary Material, Figs S4 and S5). Although this result suggests that CEP120 is required for maintaining the normal number of centrioles and centrosomes, future analysis will be required to determine the mechanism by which the *CEP120* mutation in this study causes this cellular phenotype. Interestingly, this is very similar to the centrosomal phenotype we previously described in another skeletal ciliopathy caused by deficiency of POC1A, another core centriolar protein (27).

### Knockdown of *CEP120* in zebrafish causes ciliopathy phenotypes

**Figure 4.**
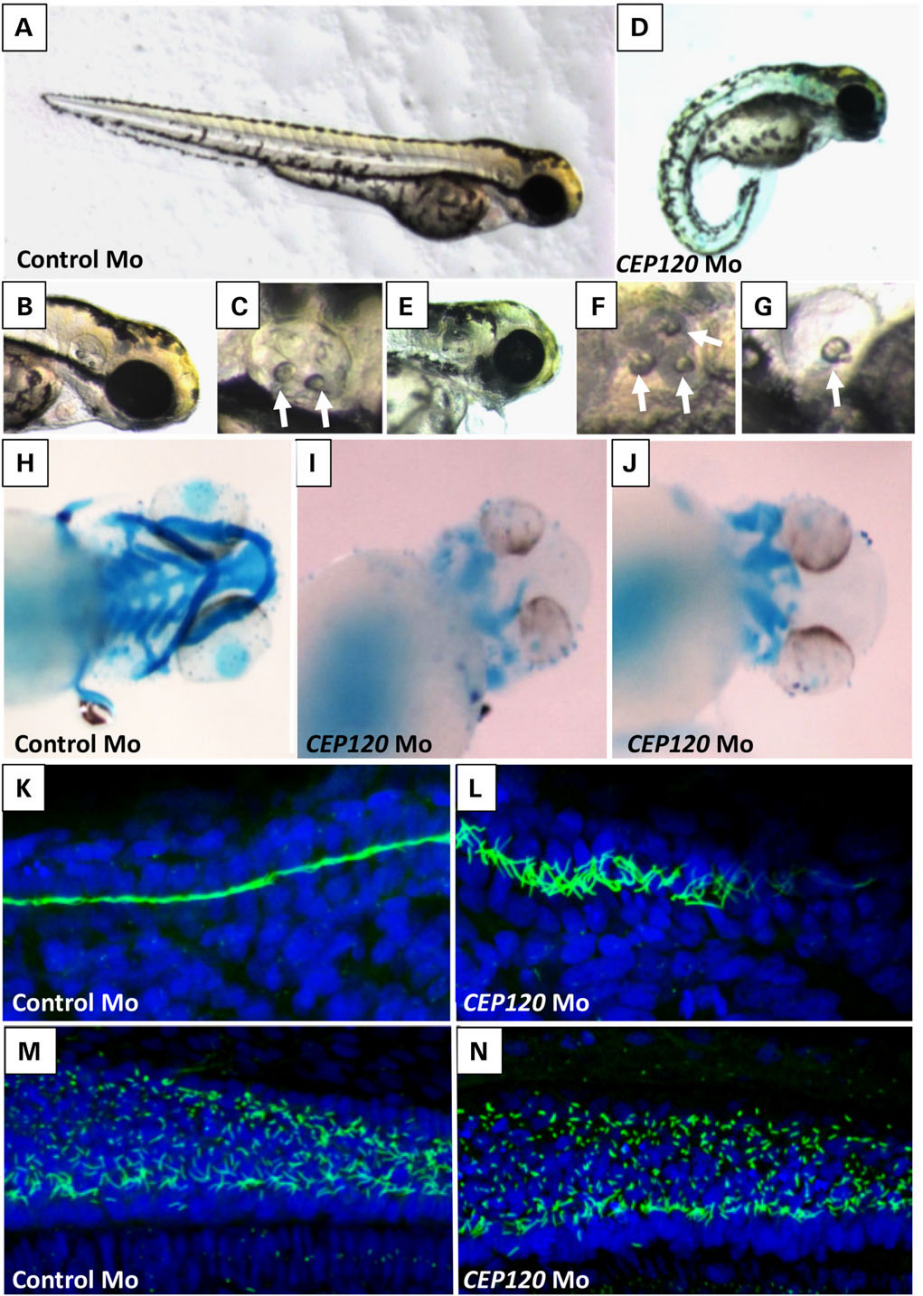
Knockdown *CEP120* ortholog in ZF results in typical ciliopathies associated phenotypes in ZF. Injection of *CEP120* antisense morpholino oligonucleotide resulting in centrally curved tail (**D**), hydrocephalus (**E**), otolith defects (**F** and **G**) and craniofacial defects (**I** and **J**) compared with control morpholino-injected embryos (**A**, **B**, **C** and **H**). Visualization of cilia using anti-acetylated tubulin in *CEP120*-injected embryos showing disorganized cilia in the pronephros (**L**) and shorter cilia in the neural tube (**N**) compared with controls (**K** and **M**).

To confirm the role of CEP120 *in vivo*, we next performed knockdown of the zebrafish (ZF) homolog (CABZ01084007.1–201, ENSDART00000122378) using an antisense morpholino oligonucleotide targeting the inton1–exon2 splice site resulted in a typical ciliopathy phenotype with ventrally curved body axis, hydrocephalus, otolith defects, heart edema and smaller eyes at 3 days post-fertilization compared with control morpholino-injected embryos (Fig. 4A–G; Supplementary Material, Fig. S6A). Embryos that survived to post-fertilization day 4 developed pronephric duct dilatation and glomerular cysts and severe general edema (Supplementary Material, Fig. S6B and C), indicating that the expanded pronephros observed at 24 h post-fertilization is likely a result of cilia dysfunction and/or cilia disorganization in the pronephros.

Alcian-Blue staining revealed striking craniofacial defects with most of the craniofacial cartilage missing (Fig. 4I and J). Co-injection of p53 morpholino did not change this phenotype (data not shown). Co-injection of human *CEP120* RNA resulted in partial but significant rescue of the curvature of the tail and hydrocephalus aspects of the morpholino-induced ciliopathy phenotype, however only the tail curvature rescue was still significant after Bonferroni correction. Co-injection of the mutant CEP120 RNA was weaker in rescue than WT RNA (significantly less rescue of the tail curvature, also after Bonferroni correction) suggesting a hypomorphic nature of the mutation (Supplementary Material, Figs S7 and S8).

We next performed immunofluorescence analysis of cilia in the pronephros and neural tube at 24 h post-fertilization. Imaging using confocal microscopy revealed disorganized cilia in the pronephric duct and shorter cilia in the neural tube (Fig. 4L and N and Supplementary Material, Fig. S9). However, we observed no significant reduction in the number of cilia (Supplementary Material, Fig. S9).

Zebrafish embryos at 24 hpf were investigated for abnormal number of centrosomes by immunofluorescence and whole-mount confocal microscopy using anti-gamma tubulin antibody (GTU88, Sigma) and nuclear counterstain (28). We did not observe multiple centrosomes in the ZF morphants or cells with increased numbers of centrioles; however, the number of cells which can be confidently assessed for the number of centrosome in the tail area is low and cells in other areas of the embryo such as the brain and pronephros could not be investigated. Therefore, the presence of cells with abnormal number of centrosomes could not be ruled out. It is possible that CEP120 loss has different consequences *in vivo* versus *in vitro*, which may explain our failure to identify cells with abnormal number of centrosomes. One can also invoke the presence of residual CEP120 protein in the embryos due to incomplete morpholino-mediated knockdown or the maternal mRNA as another explanation.

## Discussion

Most of the genes implicated in the pathogenesis of JATD are involved in IFT, but there are precedents for genes encoding resident centriolar proteins being mutated in skeletal ciliopathies and in JATD specifically. For example, a form of primordial dwarfism was found to be caused by mutations in the gene encoding the major centriolar protein POC1A (27). Very recently, we and others have shown that mutations in *CSPP1*, a gene encoding a core centriolar protein, can underlie a range of ciliopathy phenotype including JATD (16,17).

CEP120 is conserved in the flagellated unicellular organism *Chlamydomonas reinhardtii* and depletion of its ortholog Uni2 was found to eliminate formation of the flagellum (29). Subsequent analysis has demonstrated that CEP120 is also required for cilia formation in mouse tracheal epithelial cells (24). Consistent with these reports, here we show that *CEP120* knockdown in ZF embryos resulted in effects strongly resembling the phenotype observed for knockdown of other ciliary components because the cilia appeared disorganized in the pronephros and shorter in the neural tube confirming a role of CEP120 in ciliogenesis. However, in contrast to fibroblasts from affected individuals, the total number of cilia appeared normal which could be attributed to several factors as discussed above. As mouse models exhibiting strong ciliogenesis defects *in vivo* seem to be lethal around midgestation, it seems doubtful that the subjects described here who survived until term would exhibit such defects *in vivo*.

The primary cilium has an established role in skeletal development and abnormal cilia function has been associated with previously reported JATD-related genes (8,14,15,19). It seems likely that *CEP120* mutation identified in this study can cause a JATD phenotype by suppressing normal ciliary function. One proposed mechanism for how cilia mediate normal chondrogenesis and skeletal specification is through their role as signaling centers that harbor key receptors and facilitate their cascade effect in the cell, particularly, Indian hedgehog (IHH) and Sonic Hedgehog. IHH deficiency in mice results in a phenotype highly consistent with JATD, and SHH is known to act as a morphogen that determines the patterning of the limbs (30,31). Hedgehog pathway receptors localize to the cilium so it is conceivable that the pathogenesis of skeletal ciliopathies, including JATD, results from impaired hedgehog signaling as a result of lack of ciliogenesis or cilia dysfunction.

Some authors have attempted to draw clinical significance from genotype–phenotype correlation in JATD. For example*, IFT140* and *IFT172* mutations are associated with a mild thorax phenotype but a very high frequency of cystic and nephronophthisis-like renal changes and childhood-onset retinal degeneration, whereas *DYNC2H1* mutations appear to be frequently devoid of associated extra-skeletal findings except perhaps for retinal impairment detected by ERG with no reported clinically relevant visual impairment (3,8,9). Unfortunately, the very small number of individuals reported here carrying causative *CEP120* mutations does not allow for any definitive correlation although it is worth highlighting that polydactyly, which is only seen in a minority of JATD individuals, in general was observed in all four individuals in this study (Fig. 1). Furthermore, all affected individuals died from respiratory failure and a severe short rib phenotype which is not observed in individuals with *IFT140*, *IFT172* or *IFT80* mutations. Because of their short life span we cannot tell from the individuals in our study if loss of CEP120 function can be associated with clinically relevant extra-skeletal disease such as renal, hepatic or retinal involvement; however, we observed increased renal echogenicity on postnatal ultrasound in individual II:1 in Family 3. Finally, the finding of hypoplastic cerebellum in the index in Family 3 and molar tooth sign in the index in Family 4 suggests a significant overlap with oral-facial-digital syndrome type VI and Joubert syndrome. It will be interesting, therefore, to examine the contribution of *CEP120*, if any, to the mutation burden in these and other related ciliopathies.

## Conclusion

In summary, we describe a new JATD locus which we show is linked to a single mutation in a gene encoding the core centrosomal protein CEP120. This is the second time a core centrosomal protein has been implicated in the pathogenesis of JATD and we suggest that future search for candidate genes for this disease should expand beyond IFT-related genes to include others that encode proteins with other cilia-related roles.

## Materials and Methods

### Human subjects

Diagnosis of JATD was made clinically and radiologically. Affected individuals and their available relatives were recruited with informed consent according to an IRB-approved protocol.

### Autozygosity mapping

DNA samples from both affected and unaffected members were genotyped on an Axiom Chip platform as per the manufacturer's protocol (Affymetrix, Santa Clara, CA, USA). ROH >2 Mb and that span 107 SNPs were used as surrogates of autozygosity given the consanguineous nature of the families using AutoSNPa and AgileMultiIdeogram (20). The entire set of autozygosity blocks (autozygome) was determined for each patient and potential overlap between the autozygome of affected members was pursued. Shared ancestral haplotype was confirmed manually from the SNP files.

### WES

Exome capture was performed using TruSeq Exome Enrichment kit (Illumina) following the manufacturer's protocol. Samples were prepared as an Illumina sequencing library, and in the second step, the sequencing libraries were enriched for the desired target using the Illumina Exome Enrichment protocol. The captured libraries were sequenced using Illumina HiSeq 2000 Sequencer. The reads are mapped against UCSC hg19 (http://genome.ucsc.edu/) by Burrows-Wheeler aligner (http://bio-bwa.sourceforge.net/). The SNPs and Indels are detected by SAMTOOLS (http://samtools.sourceforge.net/).

### Tissue culture, ciliogenesis and immunofluorescence

For analysis of centrioles and ciliogenesis patient (individual II:2 in Family 1) and control skin fibroblasts were plated onto coverslip, cultured in Dulbecco's modified Eagle's medium (DMEM) supplemented with 10% fetal bovine serum, glutamine and penicillin and streptomycin for 48 h. The medium was replaced with OptiMem (GIBCO) without serum and cultured for 72 h. Cells were fixed in 3.6% paraformaldehyde (PFA) for 10 min, then permeabilized with 0.1% Triton X-100 (Sigma) followed by blocking in goat serum and then stained with antibodies [anti-acetylated alpha tubulin (SigmaT7451), and/or anti-pericentrin (Abcam, ab28144 and ab4448), CEP164 (Sdix cat 4533.00.02) and IFT88 (Proteintech 13967-1-AP)]. The unbound antibody was washed with phosphate buffer saline (PBS) and incubated with fluorescent-dye-conjugated secondary antibodies (ImmunoPure, Thermo Scientific). The coverslips were mounted in VectaShield containing 4′,6-diamidino-2-phenylindole (DAPI) (Vector Labs) and cells were observed under a fluorescent microscope (Nikon Eclipse 90i).

Three coverslips for each of cell lines were imaged with a fluorescent microscope. For frequency analysis, more than 100 tubulin-stained cilia were counted from each cell line.

### Zebrafish husbandry, knockdown, phenotype analysis and rescue experiments

Zebrafish husbandry was performed under standard conditions in accordance with UK Home Office and European regulations as previously described (32,33). Knockdown of the ZF homolog (CABZ01084007.1–201, ENSDART00000122378) was performed using an antisense morpholino oligonucleotide targeting the inton1–exon2 splice site (*cep120* MO: 5′-CTGACGACCTGAAGAAATACAACAA-3′). Morpholinos were dissolved in double distillation water and injections performed at the 1–4 cell stage, using standard techniques previously described (32,34). Silencing control morpholino not targeting any known ZF gene was used to control for non-specific effects due to the injection (control MO: 5′-TAGTGCAAAGCTTA-3′). p53 activation-dependent effects were excluded using 5 ng of anti-p53 morpholino (*p53* MO: 5′-GCGCCATTGCTTTGC-3′) (33). Morpholino effects on the transcript level was assessed by reverse transcription. The aberrant transcript created by the morpholino is likely degraded by nonsense medicated decay as we did not observe any band from the reverse transcription polymerase reaction experiment (Supplementary Material, Fig. S10).

A dose of 1 nl of the 0.5 mm CEP120 splice site targeting morpholino solution was used for injection. Alcian-Blue staining was performed as previously described at 4.5 days post-fertilization (33). Rescue experiments were performed using co-injection of human *CEP120* RNA synthesized from *CEP120* cDNA derived from control fibroblasts individual II:2 in Family 1 using mMessage mMachine kit (T7 Polymerase, Ambion). Scoring the ZF phenotypes was unblinded. Statistical analysis was performed using ordinary one-way ANOVA test, GraphPad Prism software.

For immunofluorescence analysis of cilia in the pronephros and neural tube at 24 h post-fertilization, ZF embryos were fixed in 4% PFA, treated with 0.05% Triton-X100 for 30 min, blocked 1 h using 4% BSA in PBS and then incubated with mouse monoclonal anti-acetylated tubulin IgG2b, clone 6-11-b1 form Sigma followed by goat anti-mouse IgG2b Alexa Fluor 488 and DAPI (Molecular Probes, Invitrogen). Imaging was carried out using a Zeiss LSM710 confocal microscope and statistical analysis was performed using Student's *t*-test, GraphPad Prism software.

## Supplementary Material

Supplementary Material is available at *HMG* online.

## Funding

Funding for KFSHRC was provided by DHFMR Collaborative Research Grant (FSA). Funding for UK10K was provided by the Wellcome Trust under award WT091310. M.S. is supported by an Action Medical Research UK Clinical Training Fellowship (RTF-1411), Radboud Excellence Fellowship and Radboud UMC Hypatia Fellowship, and M.S. and P.L.B. acknowledge funding from the Dutch Kidney Foundation (CP11.18). P.L.B. is a Wellcome Trust Senior Research Fellow and acknowledges funding from the European Community's Seventh Framework Programme FP7/2009 under grant agreement no: 241955, SYSCILIA. P.L.B. and H.M.M. are supported by the Great Ormond Street Hospital Children's Charity. E.L. is supported by a grant from the German Ministry for Education and Research (BMBF, FACE consortium, agreement no 01GM1109A, TP1) and by the European Commission (SYBIL consortium, FP7 grant agreement no. 602300 and InterregIV, project A27). H.M.M. is supported by grants from the Milena Carvajal Pro-Kartagener Foundation, Action Medical Research (GN2101) and Newlife Foundation for Disabled Children UK (10-11/15). G.J. is supported by a stipend from Association Française Contre les Myopathies. Funding to pay the Open Access publication charges for this article was provided by the Wellcome Trust, Member of the Charity Open Access Fund (CAOF).

## Supplementary Material

Supplementary Data
