# Supplementary material for: A founder CEP120 mutation in Jeune asphyxiating thoracic dystrophy expands the role of centriolar proteins in skeletal ciliopathies
Source: Hum Mol Genet. 2014 Oct 30;24(5):1410–9. doi: 10.1093/hmg/ddu555 (PMC4321448; doi:10.1093/hmg/ddu555)
Supplement: Supplementary Data [file supp_24_5_1410__index.html]

A Recurrent CEP120 Mutation in Jeune Asphyxiating Thoracic Dystrophy Expands the Role of Centriolar Proteins in Skeletal Ciliopathies — A founder CEP120 mutation in Jeune asphyxiating thoracic dystrophy expands the role of centriolar proteins in skeletal ciliopathies — A founder CEP120 mutation in Jeune asphyxiating thoracic dystrophy expands the role of centriolar proteins in skeletal ciliopathies — Supplementary Data 

# A founder *CEP120* mutation in Jeune asphyxiating thoracic dystrophy expands the role of centriolar proteins in skeletal ciliopathies

## Supplementary Data

Supplementary Data

**Files in this Data Supplement:**

- Supplementary Data - Pdf file
- Supplementary Table - xlsx file
