# Supplementary material for: A founder CEP120 mutation in Jeune asphyxiating thoracic dystrophy expands the role of centriolar proteins in skeletal ciliopathies
Source: Hum Mol Genet. 2014 Oct 30;24(5):1410–9. doi: 10.1093/hmg/ddu555 (PMC4321448; doi:10.1093/hmg/ddu555)
Supplement: Supplementary Data [file supp_ddu555_ddu555supp.pdf]

Figure S1. A diagram of morphology of the primary cilium depicting the ten genes linked thus far to JATD including *CEP120*.

Figure S2. A) Genomewide linkage analysis between the four families showing a LOD score of 3.6 on chromosome 5. B) AutoSNPa showing the shared haplotype between individual II:2 in Family 1, Individual II:4 in Family 2 and Individual II:1 in Family 3 denoted by black lines (boxed in red lines).

Figure S3. Pedigree of individual II:1 in Family 3 and Individual II:1 in Family 4 and sequence chromatogram of the same missense mutation identified in the two Saudi families.

Figure S4. Immunofluorescence images and graph of serum-starved fibroblasts from individual II:2 in Family 1 and control fibroblasts stained for the ciliary markers IFT88 (Proteintech)(red), and acetylated a-tubulin (Sigma-Aldrich)(green), and DNA (blue). Compared to control, fibroblasts from II:2 in family 1 showed a marked decrease in cilium number with only 13% of cells formed cilia.

Figure S5. Immunofluorescence images of serum-starved fibroblasts from individual II:2 in Family 1 and control fibroblasts stained for CEP164 (Sdix)(red), pericentrin (Abcam)(green), and DNA (blue) showing one representative cell with increased number of centrosomes compared to control.

Figure S6. Injection of *CEP120* antisense morpholino oligonucleotide resulting in small eye (C), pronephric dilatation (B) and severe general edema (C). Surviving embryos were examined at 4 days post fertilization.

Figure S7. *CEP120* morphants rescue experiment by co-injection with human *CEP120* RNA synthesized from *CEP120* cDNA derived from fibroblasts resulted in partial rescue of the morpholino induced ciliopathy phenotype. Graphs showing quantification of tail curvature, hydrocephalus, otolith defects and death with and without the rescue experiment. The graphs were generated using one-way anova analysis for the three groups (control, morpholino and rescue) with graph pad prism program. After Bonferroni correction (p= 0.00278), only straight tail and strong tail curvature were found to differ significantly between morpholino alone and morpholino plus WT RNA and morpholino plus WT RNA versus morpholino plus mutant RNA.

Figure S8. Graphs showing quantification of tail curvature, hydrocephalus, Otolith defects and death for the *CEP120* morphants rescue experiment by co-injection with human either *CEP120* RNA synthesized from

CEP120 cDNA derived from control fibroblasts or CEP120 mutatnt RNA synthesized from CEP120 cDNA from Individual II:2 of Family 1.

Figure S9. Assessment of cilia count and length in the neural tube of morphant zebrafish. There was no significant reduction in the cilia count but they were significantly shorter in morphants compared to controls.

Figure S10. RT-PCR gel image to the *CEP120* splice site targeting morpholino showing that the aberrant transcript created by the morpholino is likely degraded. The cDNA was validated using *GAPDH*.

FigureS1

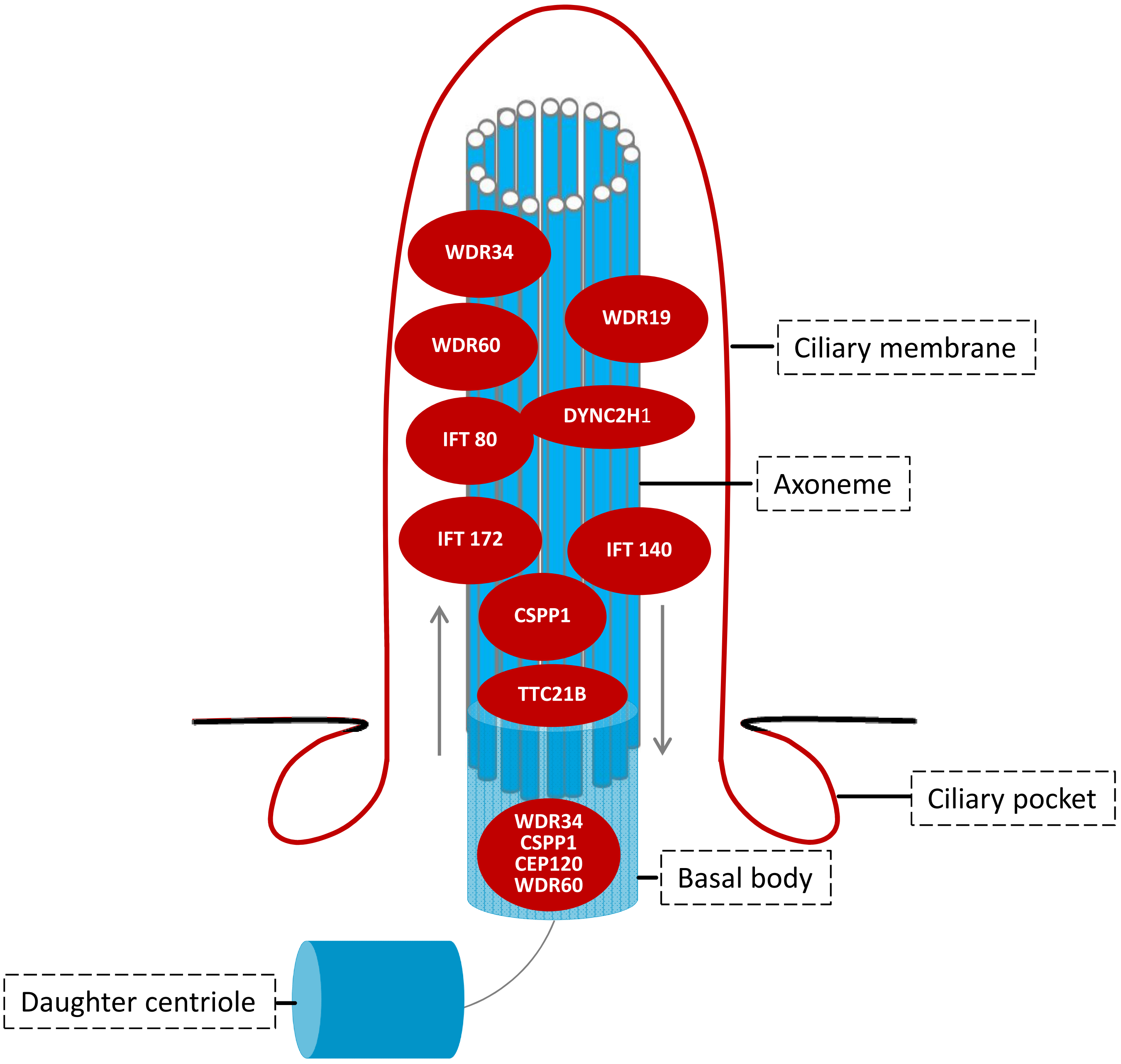
WDR34
WDR19
WDR60
DYNC2H1
IFT 80
IFT 172
IFT 140
CSPP1
TTC21B
WDR34
CSPP1
CEP120
WDR60
Ciliary membrane
Axoneme
Ciliary pocket
Basal body
Daughter centriole

Figure S2

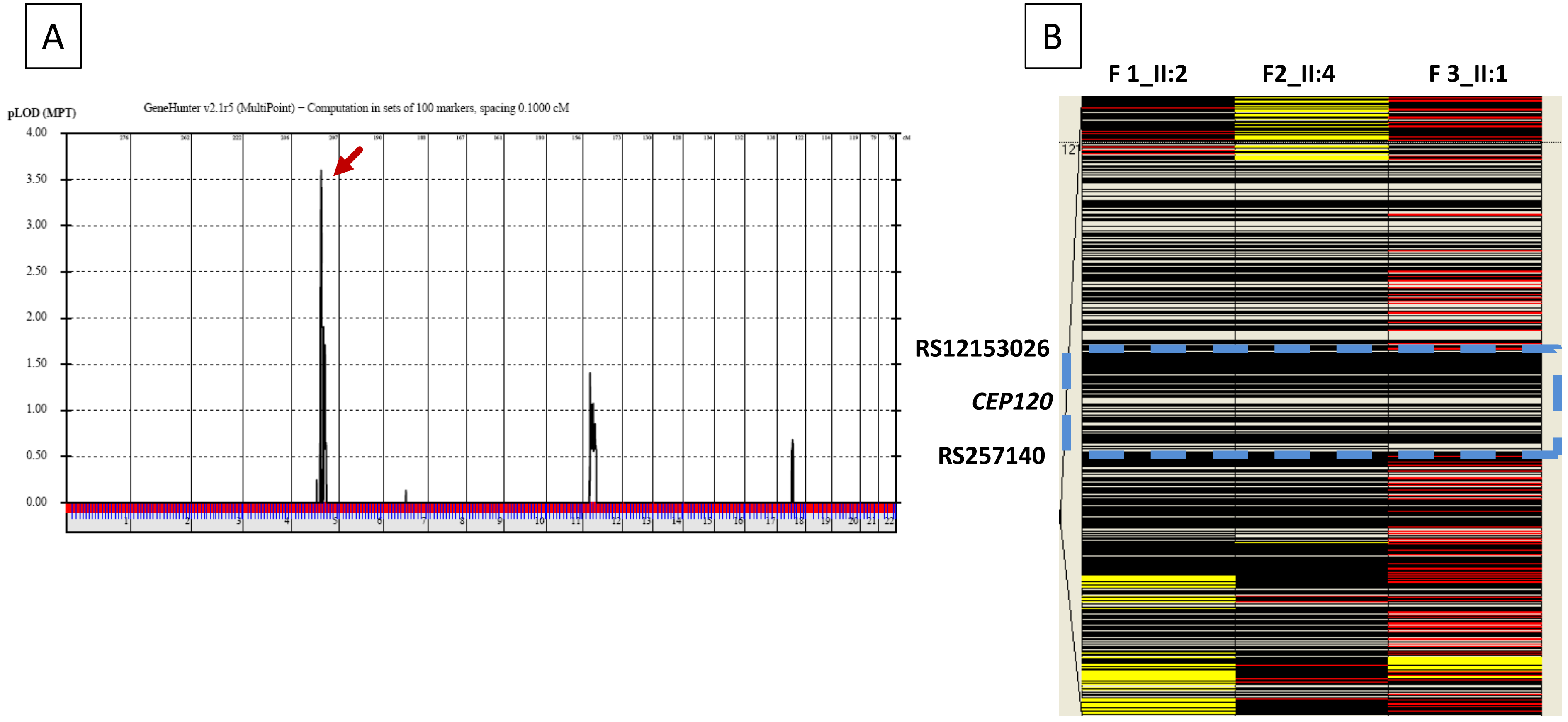

A
pLOD (MPT)
GeneHunter v2.1r5 (MultiPoint) – Computation in sets of 100 markers, spacing 0.1000 cM
4.00
3.50
3.00
2.50
2.00
1.50
1.00
0.50
0.00
B
F 1_II:2
F2_II:4
F 3_II:1
RS12153026
CEP120
RS257140

Figure S3

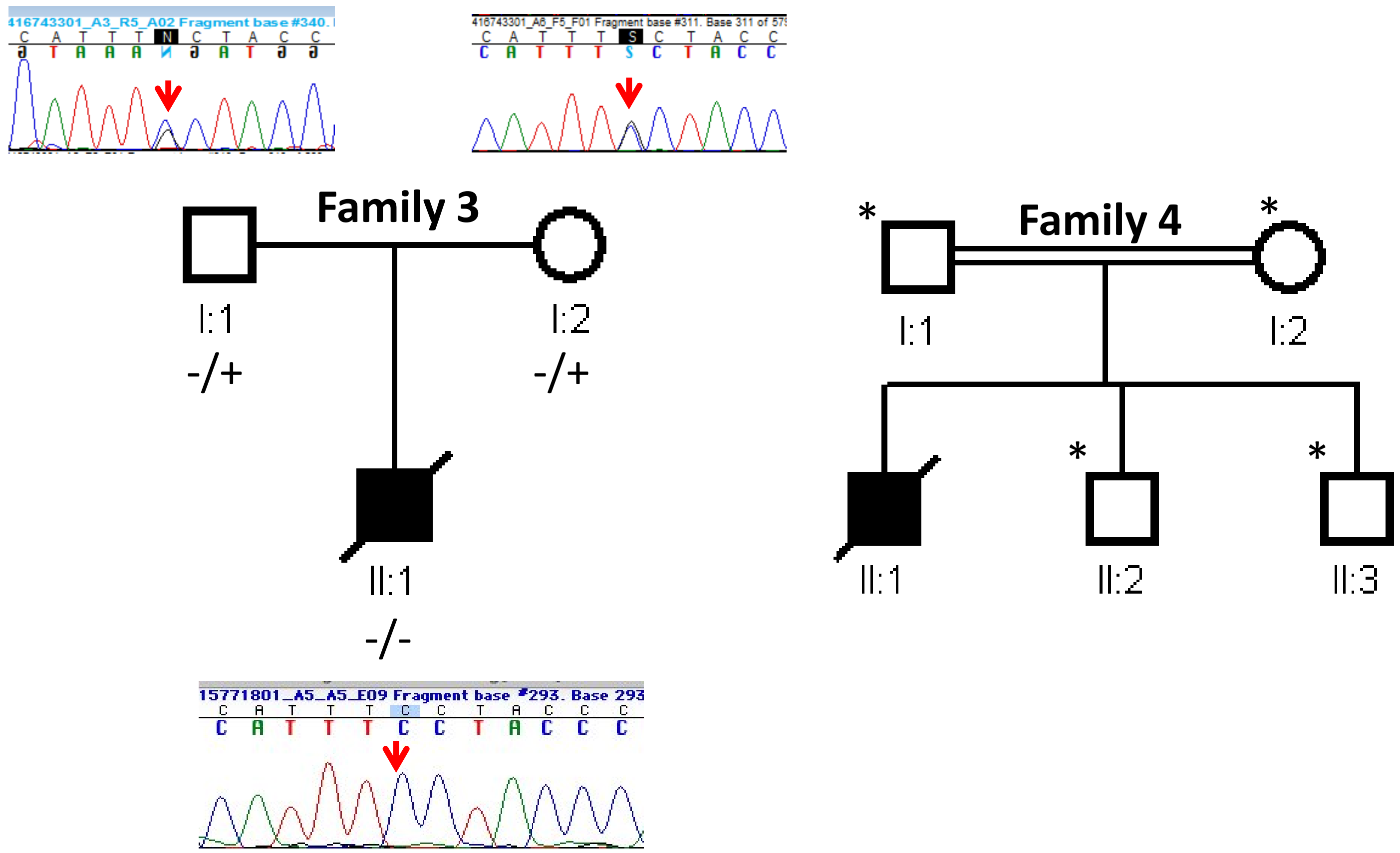

Family 3
I:1
-/+
I:2
-/+
II:1
-/-
15771801_A5_A5_E09 Fragment base #293. Base 293
Family 4
*
I:1
*
I:2
II:1
*
II:2
*
II:3

Figure S4

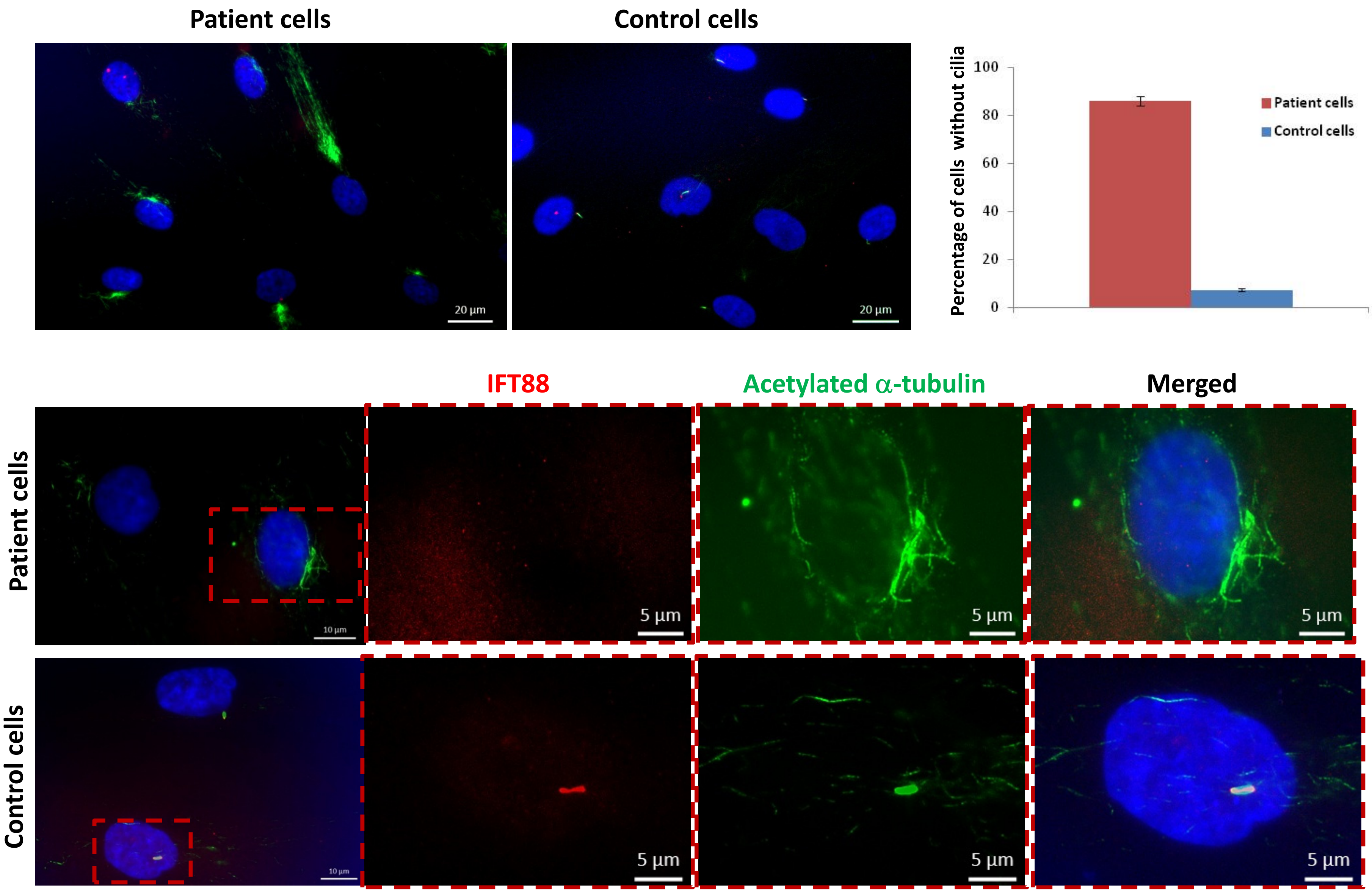
Patient cells
Control cells
20 µm
20 µm
Percentage of cells without cilia
100
80
60
40
20
0
Patient cells
Control cells
IFT88
Acetylated α-tubulin
Merged
Patient cells
Control cells
10 µm
5 µm
5 µm
5 µm
10 µm
5 µm
5 µm
5 µm

Figure S5

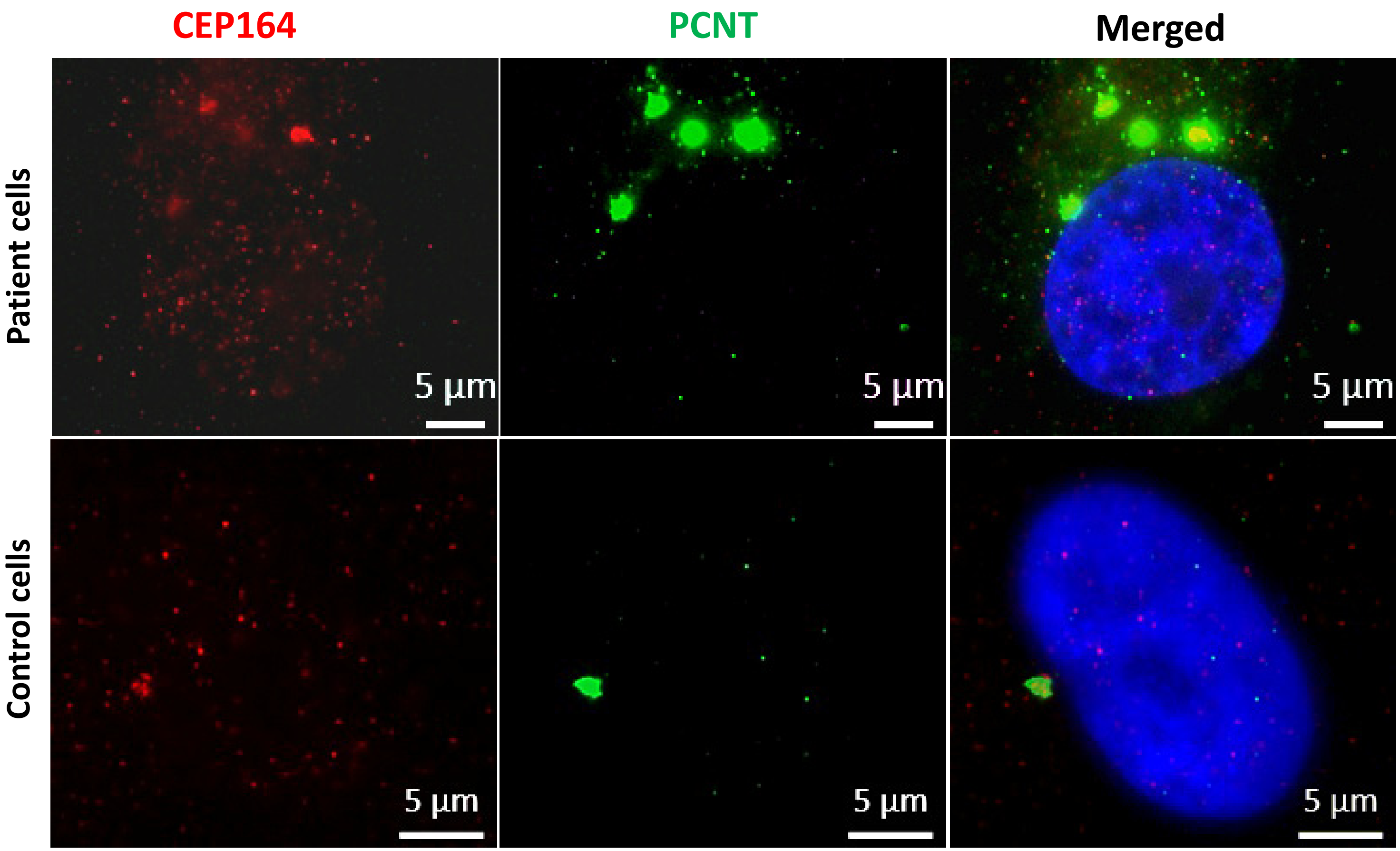
CEP164
PCNT
Merged
Patient cells
Control cells
5 µm
5 µm
5 µm
5 µm
5 µm
5 µm

Figure S6

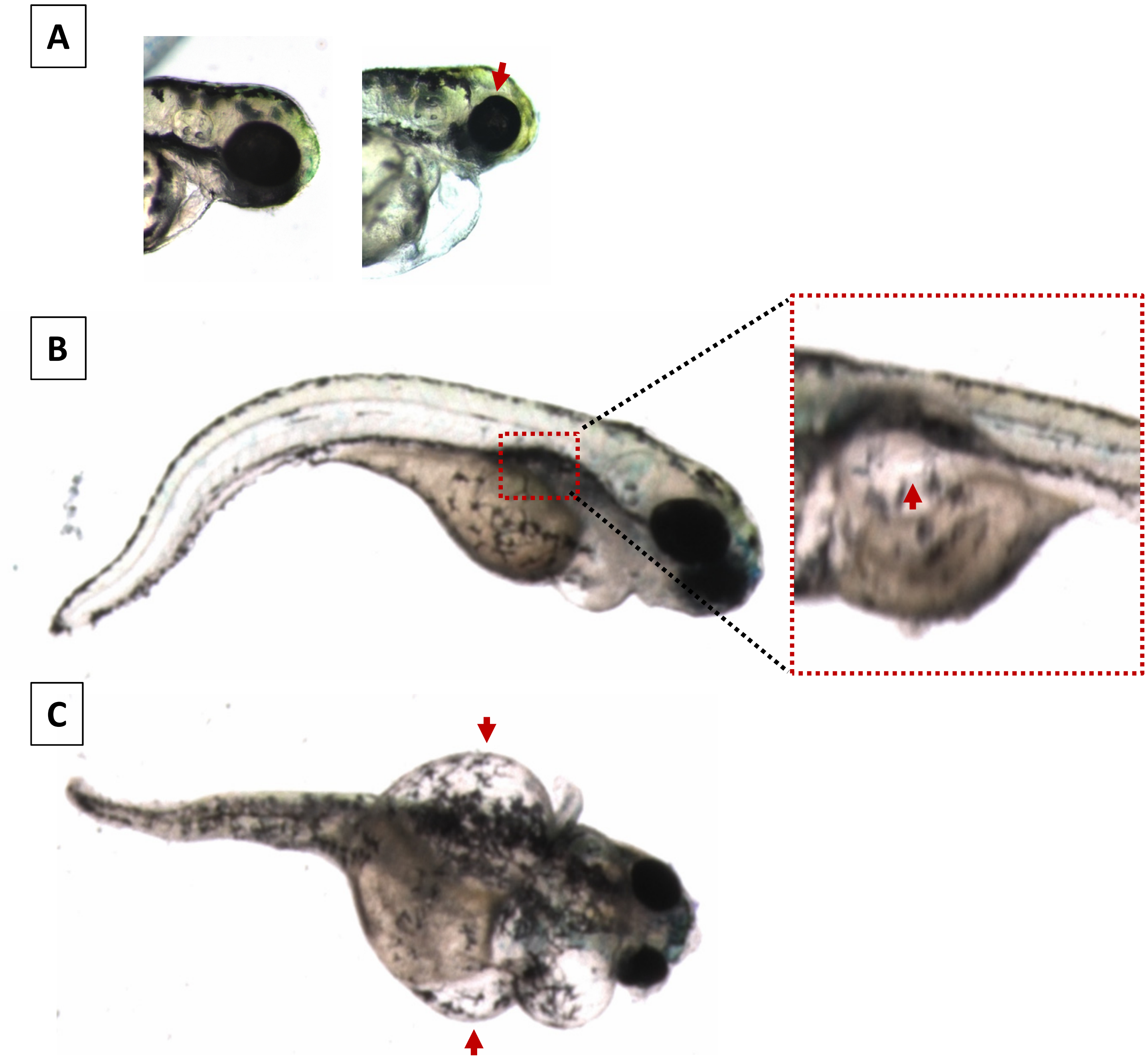

Figure S7

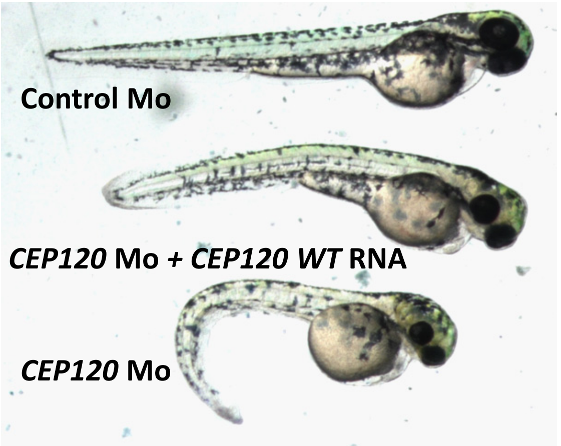

all embryos	n=107	n= 73	n= 156	n= 120	n=71	n=58	n=28	n= 30	n= 37	n=31	n=25
surviving	n=102	n=71	n=126	n=100	n= 59	n=51	n=23	n= 22	n=29	n=25	n=18
	**control 1**	**control2**	**CEP120 Mo1**	**CEP120 Mo2**	**CEP120 Mo 3**	**rescue1**	**rescue 2**	**rescue 3**	**mutant RNA rescue 1**	**mutant RNA rescue 2**	**mutant RNA rescue 3**
**straight**	100	71	16	11	9	21	11	8	6	5	4
**mild tail curvature**	2	2	36	35	14	21	9	8	13	12	6
**strong tail curvature**	0	0	74	54	36	9	3	6	10	8	8
**hydrocephalus**	2	0	47	42	28	9	3	6	4	6	6
**otolit defects**	2	2	41	29	13	6	4	5	7	6	6
**heart edema**	3	2	70	49	25	13	5	8	8	8	6
**small eyes**	1	1	93	48	27	18	6	7	9	11	7
**death**	5	2	30	20	22	7	5	9	8	5	6

Figure S8

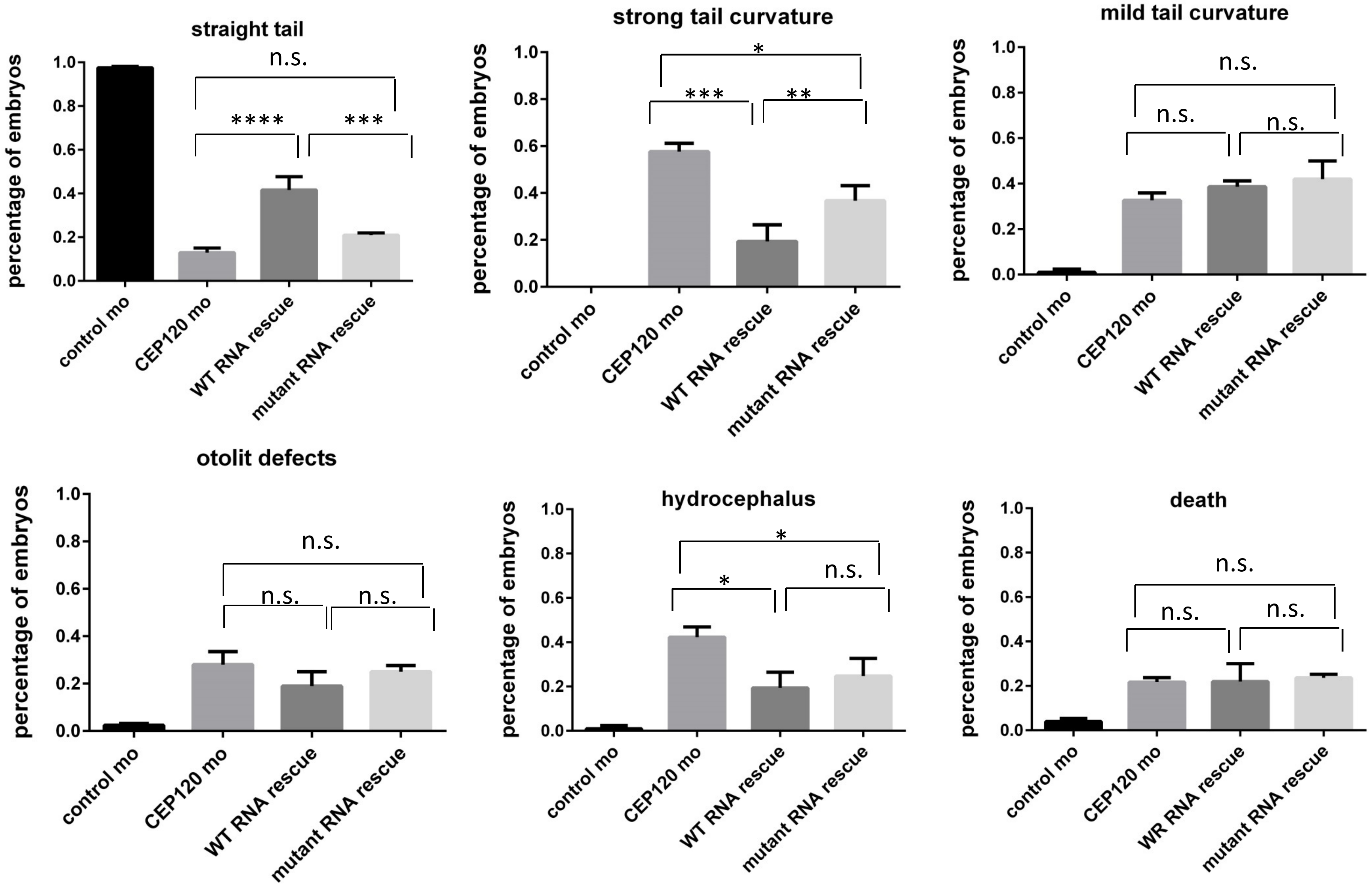

*   P<0.05   **   P<0.005   ***   P<0.0005   **** p<0.00005

Figure S9

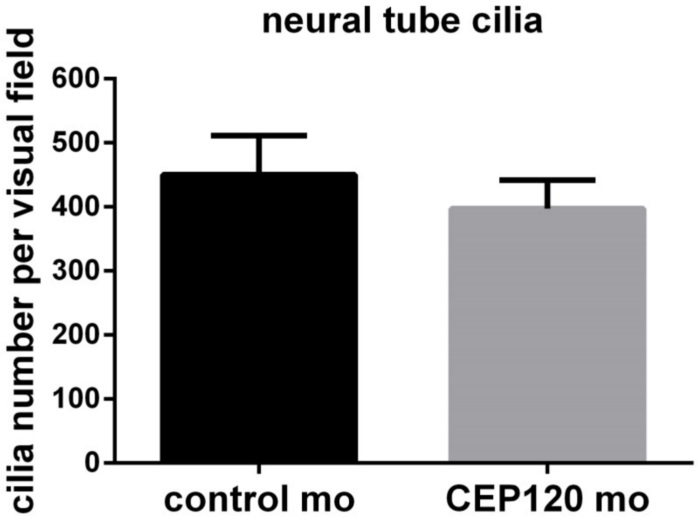

t-test using graphpad prism:
p= 0.116, non significant
Control mo 450 +/- 24.79
Cep120 mo 397 +/- 18.22

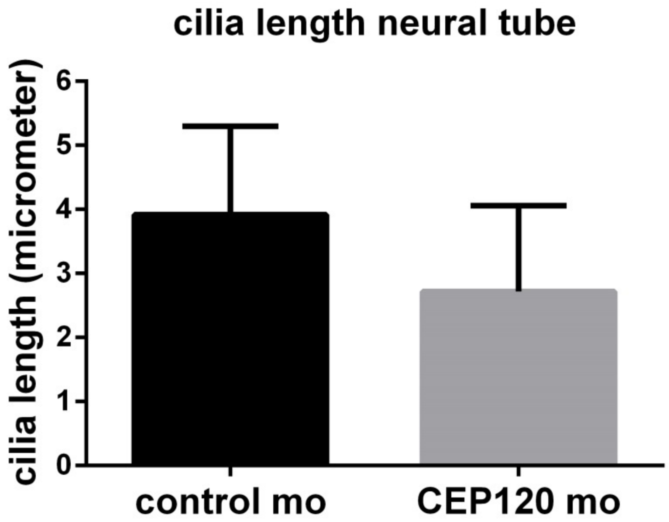

t-test using graphpad prism:
P<0.0001
Control mo 3.916 +/- 0.137
Cep120 mo 2.720 +/- 0.151

Figure S10

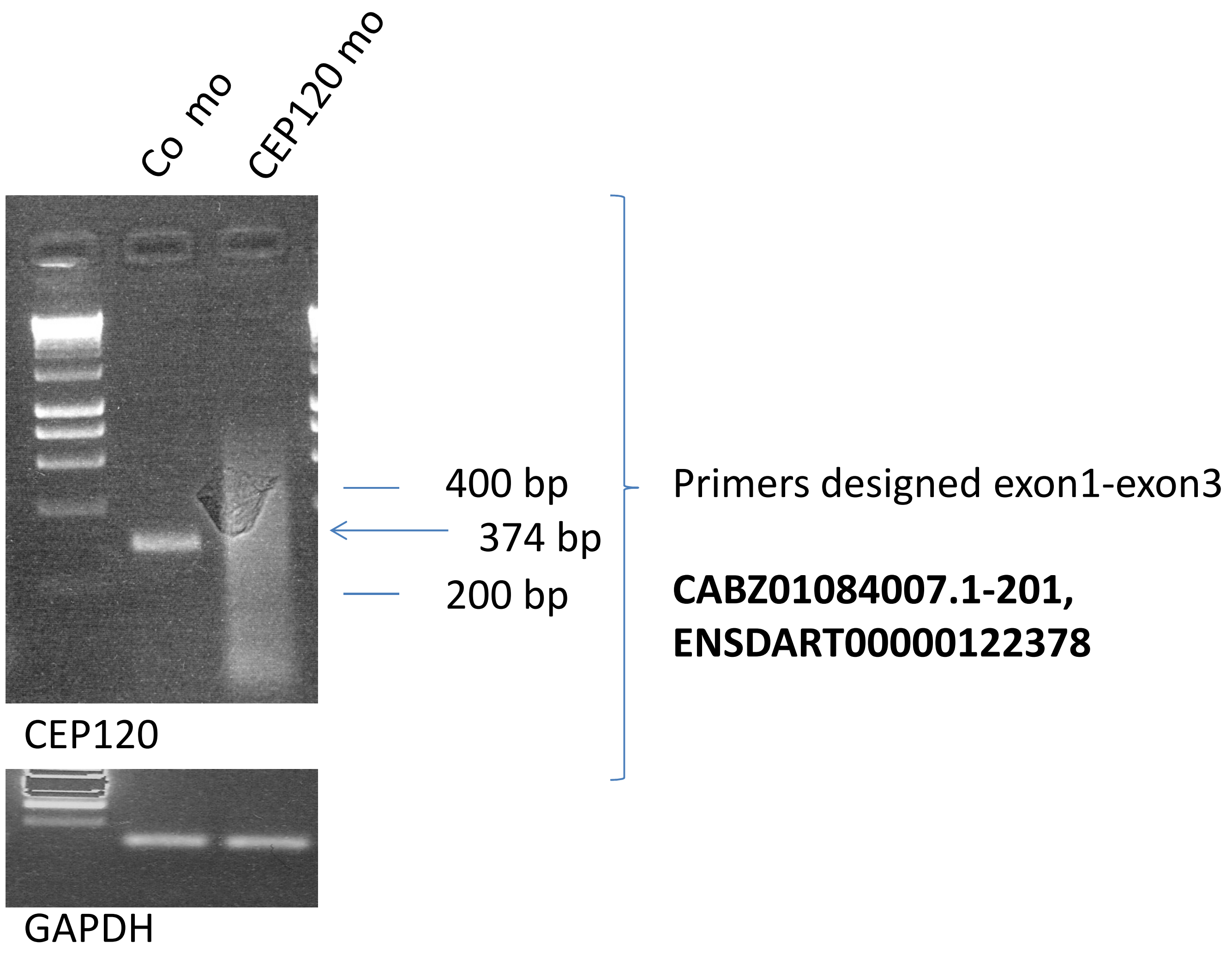
Co mo
CEP120 mo
400 bp
374 bp
200 bp
Primers designed exon1-exon3
CABZ01084007.1-201,
ENSDART00000122378
CEP120
GAPDH
